# The Inflammatory Response of Primary Bovine Mammary Epithelial Cells to *Staphylococcus aureus* Strains Is Linked to the Bacterial Phenotype

**DOI:** 10.1371/journal.pone.0087374

**Published:** 2014-01-30

**Authors:** Christina Zbinden, Roger Stephan, Sophia Johler, Nicole Borel, Julia Bünter, Rupert M. Bruckmaier, Olga Wellnitz

**Affiliations:** 1 Veterinary Physiology, Vetsuisse Faculty University of Bern, Bern, Switzerland; 2 Institute for Food Safety and Hygiene, Vetsuisse Faculty, University of Zurich, Zurich, Switzerland; 3 Institute for Veterinary Pathology, Vetsuisse Faculty, University of Zurich, Zurich, Switzerland; 4 Graduate School for Cellular and Biomedical Sciences, University of Bern, Bern, Switzerland; Indian Institute of Science, India

## Abstract

*Staphylococcus aureus* is a major mastitis-causing pathogen in dairy cows. The latex agglutination-based Staphaurex test allows bovine *S. aureus* strains to be grouped into Staphaurex latex agglutination test (SLAT)-negative [SLAT(−)] and SLAT-positive [SLAT(+)] isolates. Virulence and resistance gene profiles within SLAT(−) isolates are highly similar, but differ largely from those of SLAT(+) isolates. Notably, specific genetic changes in important virulence factors were detected in SLAT(−) isolates. Based on the molecular data, it is assumed that SLAT(+) strains are more virulent than SLAT(−) strains. The objective of this study was to investigate if SLAT(−) and SLAT(+) strains can differentially induce an immune response with regard to their adhesive capacity to epithelial cells in the mammary gland and in turn, could play a role in the course of mastitis. Primary bovine mammary epithelial cells (bMEC) were challenged with suspensions of heat inactivated SLAT(+) (n = 3) and SLAT(−) (n = 3) strains isolated from clinical bovine mastitis cases. After 1, 6, and 24 h, cells were harvested and mRNA expression of inflammatory mediators (TNF-α, IL-1β, IL-8, RANTES, SAA, lactoferrin, GM-CSF, COX-2, and TLR-2) was evaluated by reverse transcription and quantitative PCR. Transcription (ΔΔCT) of most measured factors was induced in challenged bMEC for 6 and 24 h. Interestingly, relative mRNA levels were higher (*P*<0.05) in response to SLAT(+) compared to SLAT(−) strains. In addition, adhesion assays on bMEC also showed significant differences between SLAT(+) and SLAT(−) strains.

The present study clearly shows that these two *S. aureus* strain types cause a differential immune response of bMEC and exhibit differences in their adhesion capacity in vitro. This could reflect differences in the severity of mastitis that the different strain types may induce.

## Introduction

Mastitis has a profound impact on dairy production causing considerable economic losses [1], and affecting animal welfare [2]. Its predominant cause is the invasion of bacteria that enter the udder via the teat canal [3], [4]. The virulence of the pathogen and the immune response of the cow determine the clinical outcome of mastitis [5], [6].

According to several in vivo and in vitro studies [7], distinct bacterial species affect the cow's immune response differently: *E. coli* and its cell wall component LPS induce a stronger and qualitatively different immune reaction in the mammary gland and in bovine mammary epithelial cells (bMEC) than *S. aureus* or LTA from *S. aureus*. Moreover, it was shown that different strains of a bacterial species have varying effects on the immune response [5], [8], [9].

*S. aureus* belongs to one of the most important etiological agents of bovine mastitis and is referred to as a major udder pathogen. The Staphaurex latex agglutination test (Remel, Oxoid, Pratteln, Switzerland) is a diagnostic instrument widely used to confirm putative *S. aureus* isolates through detection of characteristic *S. aureus* surface proteins. Latex particles coated with human IgG and fibrinogen interact with the bacterial target proteins SpA (staphylococcal protein A), ClfA/B (clumping factor A/B), and FnbA/B (fibronectin-binding protein A/B), mediating a rapid agglutination reaction visible to the naked eye. While the Staphaurex latex agglutination test exhibits high specificity (99.5%) and sensitivity (99.8%) when applied to *S. aureus* strains obtained from humans, Stutz et al. [10] reported that 54% of *S. aureus* isolates obtained from cases of bovine mastitis yield negative test results. The latex agglutination-negative phenotypes are due to sequence polymorphisms leading to impaired functionality of one or several of the targeted virulence factors Spa, ClfA/B, and FnbA/B. According to Moser et al. [11], all Staphaurex latex agglutination test (SLAT)-negative [SLAT(−)] strains belong to clonal complex (CC) 151, whereas SLAT-positive [SLAT(+)] strains can be assigned to various CCs. Furthermore, DNA microarray profiles for virulence and resistance genes were highly similar among SLAT(−) isolates, but differed largely from those of SLAT(+) isolates. Based on all this molecular data, it is assumed that SLAT(+) strains exceed SLAT(−) strains in virulence [11].

The bMEC lining the inner surface of the mammary gland are crucial for the early defense against intramammary pathogens. They constitute a physical barrier, and they produce, concomitantly to somatic cells in milk, several antimicrobial substances and inflammatory mediators that enhance effector functions of innate immunity and stimulate adaptive immunity [6], [12], [13].

Pathogens that enter the lumen of the mammary gland are sensed via Toll-like receptors (TLR) that recognize pathogen-associated molecular patterns (PAMPs) [14]–[16]. Finally, signaling pathways that eventually activate transcription factors for genes that promote protective and inflammatory responses are induced. Important factors of the mammary immune response include the pro-inflammatory cytokines tumor necrosis factor-α (TNF-α) and interleukin (IL)-1β, known to be expressed in the early immune response of the mammary gland [1]. They induce other immune factors like granulocyte-macrophage colony stimulating factor (GM-CSF) which mediates differentiation of hematopoietic progenitors into granulocytes and monocytes [17]. Chemokines such as IL-8 and RANTES (Regulated and on activation, normal T cell expressed and secreted) attract leukocytes from the blood to the site of infection, which is reflected by an increase of somatic cell count (SCC) in milk [1]. Furthermore, antibacterial proteins including lactoferrin (Lf) and serum amyloid A (SAA), as well as cyclooxygenase-2 (COX-2), an enzyme involved in the prostaglandin synthesis, are secreted by bMEC [12], [18], [19].

As it is assumed that SLAT(+) are more virulent than SLAT(−) and the immune response of the mammary gland to bacteria of different virulence is diverse, the aim of the present study was to compare the immune response of bMEC and their adhesion capacity to different *S. aureus* strains, belonging either to the SLAT(+) or SLAT(−) group.

According to the current study, there is clear evidence that the SLAT phenotype affects the immune response of bMEC and the adhesion capacity in vitro.

## Materials and Methods

### S. aureus strains

The six *S. aureus* strains (Table 1) used in this study were isolated from bovine mastitis milk samples collected from different cows [11]. The milk samples from mastitis were collected after the diagnosis of acute mastitis by the attending veterinarian, including a positive result of the California Mastitis Test. Bacterial cultures were maintained in peptone with 20% glycerol at −70°C. Prior to experiments, isolates were grown overnight on tryptic soy agar (TSA; Difco, Becton Dickinson Diagnostics, Mississauga, ON, Canada) at 37°C. Individual *S. aureus* strains were then inoculated in 10 ml cultures of brain heart infusion (BHI; Oxoid, Pratteln, Switzerland) broth and grown overnight at 37°C. Colony counts were determined for each strain (∼10^9^ CFU/ml).

**Table 1:** *S. aureus* strains used in this study.

Strain	Clonal complex	Staphaurex test phenotype	Anamestic data
692	CC8	positive	acute mastitis
1130	CC97	positive	acute mastitis
1989	CC20	positive	acute mastitis
1586	CC705	negative	acute mastitis
1904	CC705	negative	acute mastitis
2071	CC705	negative	acute mastitis

Reference: [11].

### Treatment of bMEC with different *S. aureus* strains

Primary cultures of mammary gland epithelial cells of two Holstein dairy cows with clinically healthy udders (SCC<10^5^ cells/ml) were developed as previously described [20]. Mammary tissue was removed directly after slaughter with permission of the slaughterhouse Marmy SA, Estavayer-le-Lac, Switzerland. Cells in passage 2 were cryopreserved in DMEM/F12 (Sigma-Aldrich, Munich, Germany) containing 20% fetal bovine serum (FBS, Sigma-Aldrich) and 10% dimethyl sulfoxide (DMSO, Sigma-Aldrich) and stored in aliquots at −80°C until the experiment. Cells from both cows were thawed and cultured in growth medium consisting of DMEM/F12 supplemented with 10% FBS, penicillin G (500 units, Sigma-Aldrich), streptomycin (100 µg/ml, Sigma-Aldrich), and ITS (0.5 mg/ml insulin, 0.5 mg/ml apo-transferrin, 0.5 µg/ml sodium selenite; Sigma-Aldrich). After two further passages, cells were seeded at a concentration of 3×10^5^ cells/well on BD Falcon™ 6-well cell culture plates (BD Biosciences, San Jose, CA, USA). On the following day, growth medium was replaced by DMEM/F12 supplemented with 5% FBS and ITS.

Suspensions of heat inactivated bacteria (inactivated by heating for 20 min at 80°C) were diluted to a concentration of 1×10^9^ CFU/ml in BHI broth. The cells were challenged in triplicate with 150 µl of the bacterial suspension. Assuming that 3×10^5^ cells/well after 24 h of incubation and a confluence of about 70% represent approximately 1×10^6^ cells/well, this corresponds to a multiplicity of infection (MOI) of 150. As a positive control, cells were challenged with 150 MOI of *Escherichia coli* in duplicate. Cells incubated in DMEM/F12, 5% FBS, and ITS only served as negative controls. Cells were incubated at 37°C with 5% CO_2_.

### Total RNA extraction and reverse transcription

After 1, 6, or 24 h of incubation, cells were harvested with 0.5 ml peqGOLD Trifast™ (PEQLAB Biotechnologie GmbH, Erlangen, Germany) and total RNA was extracted according to the manufacturer's protocol. Total RNA yield and purity were determined by absorbance at 260 nm and 280 nm using a NanoDrop-2000 spectrophotometer (Thermo Fisher Scientific Inc., Waltham, MA, USA). Finally, 500 ng of total RNA was reverse transcribed by 200 units of Moloney Murine Leukemia Virus Reverse Transcriptase (M-MLV RT; Promega Corp., Madison, WI, USA) using 100 pmol of random hexamer primers (Invitrogen, Leek, The Netherlands).

### Quantitative real-time PCR

Quantitative real-time PCR analysis was performed with the Sensimix DNA Kit (Quantace, Biolabo, Châtel St. Denis, Switzerland) on a Rotor-Gene 6000 (Corbett Research, Sydney, Australia). One reaction mixture contained 2 µl of cDNA equivalent to 25 ng of total RNA, 0.8 µl RNase-free water (Qiagen, Hilden, Germany), 1 µl (5 pmol) of forward primer, 1 µl (5 pmol) of reverse primer, and 5.2 µl of 2x SensiMix plus SYBR-Green (1 mM MgCl_2_). Primers for the housekeeping (GAPDH and ubiquitin) and target genes were synthesized commercially (Microsynth, Balgach, Switzerland) using previously published sequences [12], [18], [21], [22], or designed using the open source primer design software Primer 3 (primer sequences are listed in Table 2).

**Table 2:** Sequences, accession numbers, annealing temperature of the PCR primers, and length of the PCR products.

Gene^1^	Sequence 5′→3′		GenBank accession no.	Annealing temperature (°C)	Length (bp)
IL-1β	for	AGT GCC TAC GCA CAT GTC TTC^2^	M37211	60	114
	rev	TGC GTC ACA CAG AAA CTC GTC^2^			
TNF-α	for	CCA CGT TGT AGC CGA CAT C^2^	NM173966	60	155
	rev	CCC TGA AGA GGA CCT GTG AG^2^			
IL-8	for	ATG ACT TCC AAG CTG GCT GTT G^2^	AF232704	60	149
	rev	TTG ATA AAT TTG GGG TGG AAA G^2^			
RANTES	for	GCC AAC CCA GAG AAG AAG TG^2^	BC102064	60	119
	rev	CTG CTT AGG ACA AGA GCG AGA^2^			
SAA	for	CCT GGG CTG CTA AAG TGA TC^3^	AF540564	57	184
	rev	TAC TTG TCA GGC AGG CCA G^3^			
Lf	for	GGC CTT TGC CTT GGA ATG TAT^4^	L08604	62	338
	rev	ATT TAG CCA CAG CTC CCT GGA G^4^			
GM-CSF	for	TTC TCC GCA CCT ACT CGC	NM174027	62	195
	rev	GTT CTT GTA CAG CTT CAG GCG			
COX-2	for	TCC TGA AAC CCA CTC CCA ACA^5^	NM174445	62	242
	rev	TGG GCA GTC ATC AGG CAC AG^5^			
TLR2	for	CAT TCC CTG GCA AGT GGA TTA TC^2^	NM174197	62	201
	rev	GGA ATG GCC TTC TTG TCA ATG G^2^			
GAPDH	for	GTC TTC ACT ACC ATG GAG AAG G^2^	NM001034034	60	197
	rev	TCA TGG ATG ACC TTG GCC AG^2^			
Ubiquitin	for	AGA TCC AGG ATA AGG AAG GCA T^2^	NM174133	62	198
	rev	GCT CCA CCT CCA GGG TGA T^2^			

for  =  forward, rev  =  reverse

1 IL-1β  =  interleukin-1β; TNF-α  =  tumor necrosis factor-α; IL-8 =  interleukin-8; RANTES  =  regulated on activation, normal T cell expressed and secreted; SAA  =  serum amyloid A; Lf  =  lactoferrin; GM-CSF  =  granulocyte-macrophage colony-stimulating factor; COX-2 =  cyclooxygenase-2; TLR2 =  toll-like receptor 2; GAPDH =  glyceraldehyde-3-phosphate dehydrogenase

Primer sequence references: ^2^ [12]; ^3^ [21], ^4^ [18], ^5^ [22].

The following 3-step PCR program was used: initial denaturation for 10 min at 95°C, followed by 40 cycles with denaturation for 15 s at 95°C, 30 s at primer-specific annealing temperature, and elongation for 20 s at 72°C. Fluorescence was acquired at 72°C after each cycle, and a dissociation melt curve of the PCR product was determined at the end of each run to verify the specificity of the PCR reactions.

Cycle threshold (Ct) values were determined by the Rotor-Gene software version 1.7.75, and the relative mRNA expression was calculated with the comparative Ct method [23] using the following equation:

ΔCt =  Ct target gene – Ct endogenous control (arithmetic mean of housekeeping genes).

To visualize the impact of SLAT(+) and SLAT(−) strains on the immune response of bMEC, data are presented as ΔΔCt± SEM, where:

ΔΔCt = ΔCt sample (1, 6, or 24 h; treated) −ΔCt negative control (1, 6, or 24 h; untreated).

### Adhesion Assay

The adhesion assay was performed as previously described [24]. Bovine mammary epithelial cells derived from the same cows mentioned above were grown on sterile plastic coverslips (13 mm diameter, Bibby Sterilin, Stone, UK) coated with rat tail collagen (BD Biosciences, Allschwil, Switzerland). The bMEC on coverslips were cultured at 37°C for 24 h in 24-well plates (24-well flat-bottom cell culture plate with Low-Evaporation Lid, TPP, Trasadingen, Switzerland) until they reached a confluency of 70–80%. Suspensions of live bacteria were used at a concentration of 150 MOI to infect coverslips in duplicate. Infected bMEC cells of both cows were incubated for 3 h at 37°C. Uninfected bMECs were incubated in parallel and used as negative controls. After 3 h post infection, cell monolayers were washed five times with Dulbecco's phosphate buffered saline (DPBS) (GIBCO, Invitrogen, Carlsbad, CA, USA) and fixed with absolute methanol (−20°C) for 10 min. After the staining with May-Grünwald-Giemsa (Fluka, Buchs SG, Switzerland), the coverslips were examined by oil immersion light microscopy at a magnification of 1000×.

Adhesion affinity of the SLAT(+) and SLAT(−) *S. aureus* strains on bMEC was assessed by counting of 200 cells per coverslip and the presence of adhered bacteria. Each cell with at least one firmly adhered bacterium was counted as positive. Mean percentage of positive cells were compared between the three SLAT(+) and the three SLAT(−) *S. aureus* strains, respectively.

### Statistical Analysis

Data are presented as means ± SEM. Statistical analysis of the inflammatory response data was performed with ANOVA using a MIXED procedure of SAS (Release 9.2; SAS Institute Inc., Cary, NC, USA). The model included strain, type, and their interaction as fixed effects. Results of the triplicates were nested within cow. Statistical analysis of the adhesion experiments was performed using an unpaired t-test. Differences were considered significant if *P*<0.05.

## Results

### Relative mRNA expression of immune factors

Cells challenged with *E. coli* (positive control) induced an increase in mRNA expression of IL-1β, TNF-α, IL-8, and GM-CSF after 1 h of stimulation. Furthermore, *E. coli* induced mRNA expression of all factors after 6 and 24 h of stimulation (data not shown).

Cells challenged with *S. aureus* for only 1 h had no significant effect on the relative mRNA expression of all measured factors in bMEC, with the exception of IL-1β and IL-8. IL-1β mRNA expression was significantly upregulated in response to most of the tested *S. aureus* strains, regardless of SLAT phenotype. IL-8 mRNA expression was significantly induced in bMEC challenged for 1 h with SLAT(+) strains (Table 3).

**Table 3:** Changes of mRNA abundance (Mean ± SEM ΔΔCt^1^) of immune factors in bMEC stimulated with six heat-inactivated *S. aureus* strains for 1, 6, or 24 h.

		SLAT(+)^3^			SLAT(−)^3^			Analysis of variance (*P*-value)		
Gene^2^	Time (h)	692	1130	1989	1586	1904	2071	Strain(Type)	Cow	Strain*Cow
IL-1β	1	0.5±0.3 ^ac^	1.6±0.2 ^b^ *	1.3±0.3 ^bc^ *	1.3±0.5 ^bc^ *	1.5±0.4 ^b^ *	0.5±0.3 ^a^	0.01	<0.01	0.03
	6	8.3±0.2 ^a^ *	8.7±0.2 ^a^ *	8.3±0.3 ^a^ *	3.5±0.1 ^b^ *	5.7±0.5 ^c^ *	5.3±0.2 ^c^ *	<0.01	<0.01	0.01
	24	5.1±0.3 ^ab^ *	5.7±0.2 ^b^ *	4.8±0.1 ^a^ *	0.4±0.4 ^c^	1.8±0.3 ^d^ *	1.6±0.3 ^d^ *	<0.01	<0.01	0.03
TNF-α	1	0.7±0.2 ^a^	0.8±0.1 ^a^	0.3±0.3 ^a^	0.2±0.6 ^ab^	05±0.5 ^a^	−0.6±0.3 ^b^	0.07	<0.01	0.02
	6	5.4±0.4 ^a^ *	5.1±0.2 ^a^ *	5.1±0.4 ^a^ *	1.3±0.6 ^b^ *	3.4±0.3 ^c^ *	2.9±0.4 ^c^ *	<0.01	<0.01	0.75
	24	4.1±0.2 ^a^ *	4.3±0.2 ^a^ *	4.1±0.1 ^a^ *	1.1±0.2 ^b^ *	2.0±0.4 ^c^ *	1.6±0.1 ^bc^ *	0.11	0.06	0.23
IL-8	1	2.0±0.5 ^a^ *	2.1±0.4 ^a^ *	1.5±0.2 ^ab^ *	0.6±0.4 ^b^	0.8±0.3 ^b^	0.9±0.5 ^b^	0.73	<0.01	0.07
	6	7.8±0.2 ^a^ *	7.1±0.2 ^b^ *	7.0±0.4 ^b^ *	4.7±0.1 ^c^ *	6.1±0.3 ^d^ *	5.4±0.3 ^e^ *	<0.01	<0.01	0.01
	24	7.1±0.3 ^a^ *	7.0±0.2 ^a^ *	7.1±0.1 ^a^ *	3.5±0.4 ^b^ *	4.8±0.2 ^c^ *	4.1±0.6 ^c^ *	<0.01	<0.01	0.01
RANTES	1	−0.2±0.2 ^ab^	−0.6±0.3 ^ab^	0.1±0.5 ^a^	−0.6±0.4 ^ab^	−0.5±0.4 ^ab^	−0.8±0.5 ^b^	0.56	<0.01	0.72
	6	3.9±0.2 ^a^ *	4.3±0.2 ^a^ *	2.9±0.3 ^b^ *	0.0±0.2 ^c^	1.8±0.4 ^d^ *	1.0±0.3 ^e^ *	<0.01	<0.01	0.69
	24	3.2±0.3 ^a^ *	4.0±0.3 ^b^ *	3.0±0.3 ^a^ *	−0.1±0.5 ^c^	1.1±0.2 ^d^ *	0.6±0.4 ^d^	<0.01	<0.01	0.06
SAA	1	−1.0±0.5 ^a^	−0.6±0.4 ^ab^	0.8±1.0 ^c^	−0.9±0.9 ^a^	0.7±0.8 ^bc^	−0.9±0.6 ^a^	0.01	<0.01	0.50
	6	5.6±0.6 ^a^ *	4.9±0.5 ^ab^ *	4.7±0.4 ^b^ *	0.8±0.4 ^c^	3.6±0.3 ^d^ *	1.6±0.3 ^e^ *	<0.01	<0.01	<0.01
	24	5.3±0.4 ^a^ *	5.3±0.6 ^a^ *	6.3±0.5 ^b^ *	1.9±0.5 ^c^ *	3.3±0.4 ^d^ *	3.0±0.6 ^d^ *	<0.01	<0.01	0.47
Lf	1	0.7±0.2 ^a^	0.1±0.3 ^bc^	−0.1±0.2 ^d^	0.4±0.4 ^ab^	−0.7±0.4 ^d^	−0.6±0.3 ^cd^	0.01	<0.01	0.53
	6	2.6±0.4 ^a^ *	2.1±0.1 ^ab^ *	1.9±0.4 ^b^ *	0.0±0.5 ^c^	1.6±0.2 ^b^ *	0.6±0.2 ^d^	<0.01	<0.01	0.02
	24	3.9±0.5 ^a^ *	3.6±0.5 ^a^ *	4.1±0.5 ^a^ *	1.9±0.8 ^b^ *	2.4±0.4 ^b^ *	1.6±0.7 ^b^ *	0.36	<0.01	0.03
GM-CSF	1	0.6±0.2 ^a^	0.0±0.4 ^ab^	−0.5±0.5 ^b^	0.3±0.1 ^a^	0.4±0.2 ^a^	0.5±0.3 ^a^	0.07	0.01	0.16
	6	3.1±0.1 ^a^ *	3.9±0.2 ^b^ *	3.0±0.4 ^a^ *	0.0±0.3 ^c^	1.6±0.2 ^d^ *	1.9±0.3 ^d^ *	<0.01	0.71	0.04
	24	0.4±0.6 ^ab^	1.7±0.4 ^c^ *	0.5±0.4 ^a^	−1.4±0.3 ^d^	−0.7±0.4 ^bd^	−1.0±0.6 ^d^	0.06	<0.01	0.39
COX-2	1	0.5±0.3	0.3±0.2	−0.1±0.4	0.6±0.3	−0.3±0.5	0.0±0.1	0.27	0.08	0.03
	6	1.8±0.2 ^a^ *	1.8±0.4 ^ab^ *	1.5±0.3 ^abd^ *	0.2±0.1 ^c^	1.1±0.1 ^bd^ *	1.0±0.4 ^d^	0.06	<0.01	0.01
	24	0.1±0.5 ^ab^	0.8±0.2 ^b^	0.8±0.2 ^b^	−0.6±0.3 ^a^	−0.3±0.2 ^a^	−0.4±0.4 ^a^	0.58	0.59	<0.01
TLR2	1	0.0±0.3 ^a^	0.6±0.3 ^a^	0.3±0.9 ^a^	0.6±0.3 ^a^	−0.3±0.3 ^a^	−0.2±0.5 ^a^	0.57	0.05	0.95
	6	2.7±0.3 ^a^ *	1.9±0.2 ^b^ *	2.0±0.3 ^b^ *	0.4±0.4 ^c^	1.7±0.2 ^bd^ *	1.1±0.3 ^d^ *	<0.01	<0.01	<0.01
	24	2.1±0.4 ^ab^ *	2.2±0.6 ^a^ *	2.6±0.5 ^a^ *	0.9±0.4 ^c^	1.5±0.3 ^bc^ *	1.1±0.6 ^c^ *	0.23	<0.01	0.51

a–e Means within a row without common superscript letters differ (*P*<0.05).

* Means differ significantly from negative control.

1 ΔΔCt values are normalized to negative controls and corrected for the two reference genes, GAPDH and ubiquitin.

2 IL-1β =  interleukin-1β; TNF-α =  tumor necrosis factor-α; IL-8 =  interleukin-8; RANTES =  regulated on activation, normal T cell expressed and secreted; SAA =  serum amyloid A; Lf =  lactoferrin; GM-CSF =  granulocyte-macrophage colony-stimulating factor; COX-2 =  cyclooxygenase-2; TLR2 =  toll-like receptor 2.

3 SLAT(−)  =  *S. aureus* latex agglutination test negative; SLAT(+)  =  *S. aureus* latex agglutination test positive.

The relative mRNA expression of IL-1β, TNF-α, and IL-8 was significantly increased after 6 and 24 h in response to all *S. aureus* strains, except for IL-1β mRNA expression after challenge with SLAT(−) strain *1586* for 24 h. After 24 h of challenge, mRNA levels of IL-1β, TNF-α, and IL-8 were equal or lower than after 6 h (Table 3).

**Figure 1:**
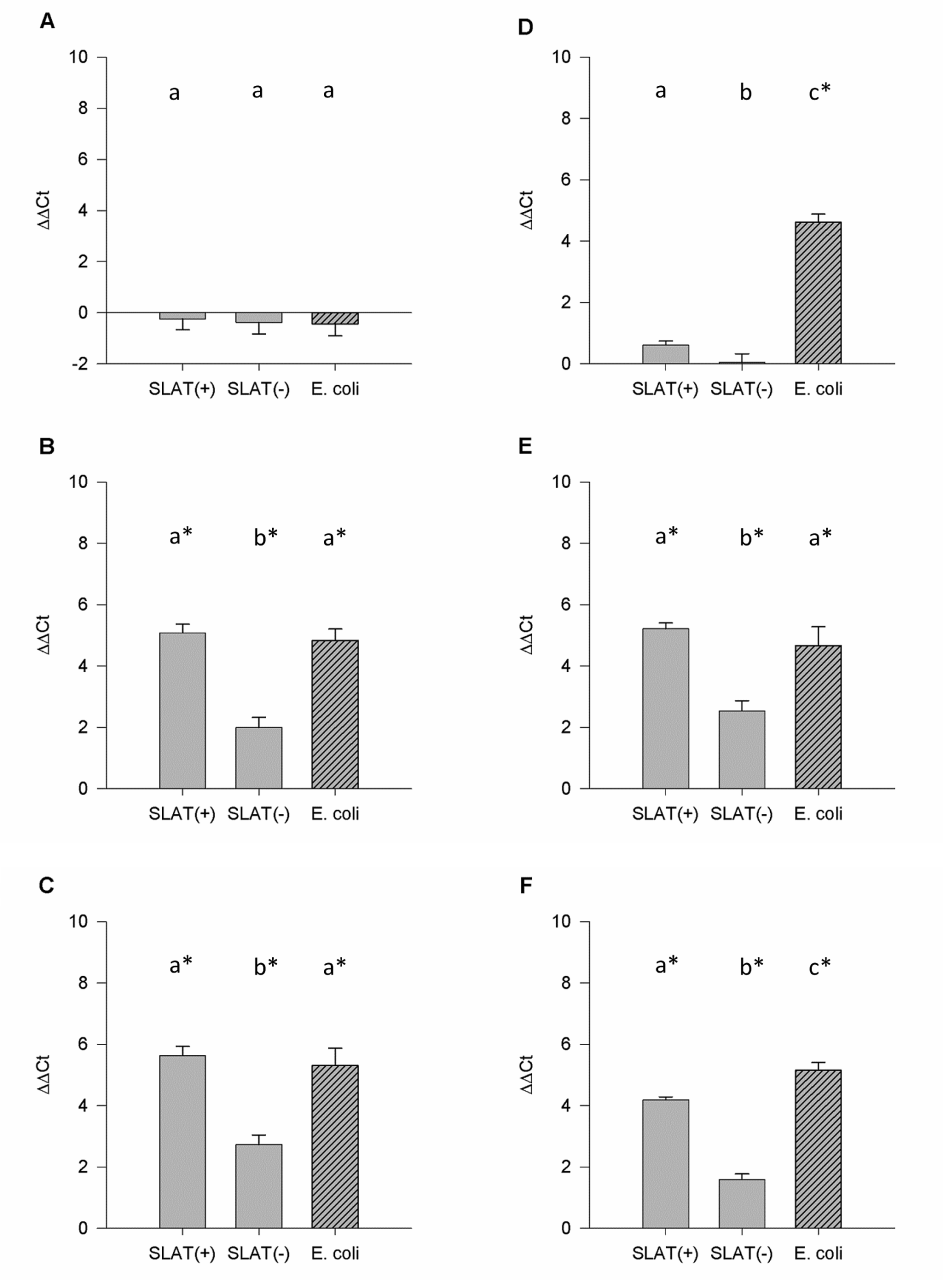
Relative SAA and TNF-α mRNA expression. Relative mRNA expression (ΔΔCt) of SAA in bMEC stimulated for (A) 1 h, (B) 6 h, or (C) 24 h with SLAT(+), SLAT(−), or *E. coli*. Relative mRNA expression (ΔΔCt) of TNF-α in bMEC stimulated for (D) 1 h, (E) 6 h, or (F) 24 h with SLAT(+), SLAT(−) or *E. coli*. Data are presented as Means ± SEM. ^a,b^Means without common superscript letters differ (*P*<0.05).

All SLAT(+) strains induced increased mRNA levels of RANTES in bMEC challenged for 6 or 24 h. Within the SLAT(−) group, only strain *1904* induced elevated mRNA levels after 6 and 24 h of challenge. For all strains, similar RANTES mRNA levels were obtained in cells challenged for either 6 or 24 h (Table 3). For IL-1β, TNF-α, IL-8, and RANTES the relative mRNA abundances were significantly higher in cells challenged for 6 and 24 h with SLAT(+) than with SLAT(−) strains (Table 4, Figure 1).

**Table 4:** Effect of SLAT type on changes of mRNA expression (Mean ± SEM ΔΔCt^1^) of immune factors in bMEC stimulated for 1, 6, or 24 h.

		Type^3^		Analysis of variance (*P*-value)	
Gene^2^	Time (h)	SLAT(+)	SLAT(-)	Type	Cow
IL-1β	1	1.1±0.2 ^a^ *	1.1±0.2 ^a^ *	0.87	0.01
	6	8.4±0.1 ^a^ *	4.8±0.3 ^b^ *	<0.01	0.03
	24	5.2±0.1 ^a^ *	1.3±0.2 ^b^ *	<0.01	<0.01
TNF-α	1	0.6±0.1 ^a^	0.0±0.3 ^b^	0.03	<0.01
	6	5.2±0.2 ^a^ *	2.5±0.3 ^b^ *	<0.01	0.02
	24	4.2±0.1 ^a^ *	1.6±0.2 ^b^ *	<0.01	0.08
IL-8	1	1.9±0.2 ^a^ *	0.8±0.2 ^b^	<0.01	<0.01
	6	7.3±0.2 ^a^ *	5.2±0.2 ^b^ *	<0.01	<0.01
	24	7.1±0.1 ^a^ *	4.1±0.3 ^b^ *	<0.01	<0.01
RANTES	1	−0.2±0.2 ^a^	−0.7±0.2 ^a^	0.09	<0.01
	6	3.7±0.2 ^a^ *	0.9±0.2 ^b^	<0.01	0.04
	24	3.4±0.2 ^a^ *	0.6±0.2 ^b^	<0.01	<0.01
SAA	1	−0.2±0.4 ^a^	−0.4±0.5 ^a^	0.52	<0.01
	6	5.1±0.3 ^a^ *	2.0±0.3 ^b^ *	<0.01	<0.01
	24	5.6±0.3 ^a^ *	2.7±0.3 ^b^ *	<0.01	<0.01
Lf	1	0.2±0.2 ^a^	−0.3±0.2 ^b^	0.05	<0.01
	6	2.2±0.2 ^a^ *	0.7±0.2 ^b^ *	<0.01	<0.01
	24	3.9±0.3 ^a^ *	2.0±0.4 ^b^ *	<0.01	<0.01
GM-CSF	1	0.0±0.2 ^a^	0.4±0.1 ^a^	0.09	0.01
	6	3.3±0.2 ^a^ *	1.2±0.2 ^b^	<0.01	0.79
	24	0.9±0.3 ^a^	−1.0±0.3 ^b^	<0.01	<0.01
COX-2	1	0.2±0.2 ^a^	0.1±0.2 ^a^	0.67	0.08
	6	1.7±0.2 ^a^ *	0.7±0.2 ^b^	<0.01	0.01
	24	0.6±0.2 ^a^	−0.4±0.2 ^b^	<0.01	0.58
TLR2	1	0.3±0.3 ^a^	0.0±0.2 ^a^	0.45	0.04
	6	2.2±0.2 ^a^ *	1.1±0.2 ^b^	<0.01	0.01
	24	2.3±0.3 ^a^ *	1.2±0.3 ^b^ *	<0.01	<0.01

a-e Means within a row without common superscript letters differ (*P*<0.05).

* Means differ significantly from negative control.

1 ΔΔCt values are normalized to negative controls and corrected for the two reference genes, GAPDH and ubiquitin.

2 IL-1β =  interleukin-1; TNF-α =  tumor necrosis factor-α; IL-8 =  interleukin-8; RANTES =  regulated on activation, normal T cell expressed and secreted; SAA =  serum amyloid A; Lf =  lactoferrin; GM-CSF =  granulocyte-macrophage colony-stimulating factor; COX-2 =  cyclooxygenase-2; TLR2 =  toll-like receptor 2.

3 SLAT(−)  =  *S. aureus* latex agglutination test negative; SLAT(+)  =  *S. aureus* latex agglutination test positive.

Lactoferrin and SAA were upregulated in bMEC challenged for 6 and 24 h with SLAT(+) strains, whereas the increase was equal or higher after 24 h (Table 3). Two of the SLAT(−) strains increased SAA mRNA expression after 6 and 24 h of stimulation (Table 3). Lactoferrin mRNA expression was significantly increased after 24 h of stimulation in response to all SLAT(−) strains, whereas after 6 h only one of the SLAT(−) strains induced Lf transcription (Table 3). SLAT(+) significantly induced higher relative mRNA levels of SAA and Lf than SLAT(−) strains (Table 4, Figure 1).

In response to all SLAT(+) strains, GM-CSF mRNA expression was significantly increased after 6 h of challenge, but decreased again between 6 and 24 h. The challenge of bMEC with SLAT(+) for 6 h caused significantly higher GM-CSF mRNA expression compared to SLAT(−) strains (Tables 3 & 4). Two SLAT(−) strains affected GM-CSF mRNA expression in bMEC stimulated for 6 h (Table 3).

Expression levels of COX-2 mRNA were affected after stimulation for 6 h with all SLAT(+), as well as with one SLAT(−) strain (Table 3). Although the SLAT(+)-induced response was significantly stronger after 6 h for COX-2 compared to SLAT(−) (Table 4), individual strain differences were not consistently significant between both groups (Table 3).

In response to all *S. aureus* strains, except for SLAT(−) *1586*, relative mRNA levels of TLR2 were significantly increased in bMEC stimulated for 6 and 24 h. The mRNA levels were similar in bMEC stimulated for 6 or 24 h (Table 3). After 6 and 24 h, the expression of TLR2 mRNA was more pronounced in bMEC stimulated with SLAT(+) than with the SLAT(−) type (Table 4).

Although SLAT(+) significantly induced higher mRNA expression levels than SLAT(−) for all measured factors (Table 4), mRNA expression differed between individual strains for most time points (Table 3). In addition, there were significant strain by cow interactions in half of the measurements, and the cells from differing cows had a significant impact on mRNA expression of all factors at most time points (Table 3). A similar expression pattern of most immune factors was seen in SLAT(+) and *E. coli* challenged bMEC after 6 and 24 h (data not shown).

### Adhesion Assay

After 3 h post infection, bMEC were morphologically unaltered irrespective of the *S. aureus* strain and when compared to the negative controls (confluency of 70–80%). All six *S. aureus* strains showed a variable degree of adhesion (10–55%) to bMEC. Clusters of more than 20 adherent bacteria were frequently observed in SLAT(+) but not in SLAT(−) *S. aureus* strains. There was a statistically significant difference in adhesion affinity between the SLAT(+) and SLAT(−) *S. aureus* strains in cells of both cows (Table 5).

**Table 5:** Degree of adhesion (%) of SLAT(+) and SLAT(−) strains to bMEC.

bMEC	SLAT(+)	SLAT(−)
cow 1	36.1±6.1 ^a^	20.6±2.8 ^b^
cow 2	36.1±6.1 ^a^	21.0±3.6 ^b^
total (cow 1 & 2)	36.3±4.9 ^a^	20.8±3.1 ^b^

a,b Means within a row without common superscript letters differ (*P*<0.05).

## Discussion

Cultured primary bMEC responded to the challenge with heat inactivated bacteria by upregulation of genes relevant in the immune reaction of the mammary gland. This was highly reproducible and verifies the suitability of the model for comparison of the innate immune response to different bacterial strains. By using cells from two different cows, the biological reproducibility was proven. With some exceptions, the factor ‘cow’ had a significant effect on the results. Differences are most likely attributed to the genotype of the cows, as it was shown that bMEC from cows with genetic differences in mastitis susceptibility can show a difference in magnitude of transcription of immune factors in response to bacterial challenge [25].

*Escherichia coli* was chosen as a positive control as it often causes acute mastitis. Heat inactivated *E. coli* isolated from acute mastitis cases induced a strong immune response characterized by highly induced mRNA expression of important immune factors in mammary epithelial cells in culture [12]. This is in agreement with the present study, as the mRNA expression for all measured factors was strongly upregulated after at least 6 h post challenge.

Three different periods of challenge were used in the present study to capture the change of different immune factors that are expressed in the early and later phases of the immune response. Griesbeck-Zilch et al. [12] found that mRNA levels of IL-1β, TNF-α, and IL-8 were already increased in bMEC after 1 h of challenge with the heat inactivated *S. aureus* strain M60. Comparable results were seen in the present study with several *S. aureus* strains, which indicates that bMEC respond directly to contact with heat inactivated *S. aureus*, regardless of the bacterial type, i.e. SLAT(+) or SLAT(−). Interestingly for IL-8, a stronger increase of transcription was detectable after 1 h of challenge for SLAT(+) strains compared to SLAT(−) strains. It is apparent for the other factors that 1 h of challenge was not long enough to induce measurable changes in transcription of the selected factors. However, after challenging the cells for a longer period of time, the induction of mRNA expression of all measured immune factors was more pronounced by SLAT(+) compared to SLAT(−) strains.

In clinical mastitis caused by *S. aureus*, IL-1β and TNF-α are expressed in the early stages of the infection period, soon followed by a considerable decrease [4], [26]. It is assumed that the duration of enhanced transcription of these pro-inflammatory cytokines is not sufficient to eliminate *S. aureus* from the mammary gland, and therefore may often lead to a chronic outcome [1]. In contrast, *E. coli* typically causes a more sustained increase of these cytokines [4]. *E. coli* intramammary infections are often characterized by acute and severe clinical manifestations, but can, if not leading to death, be cured within a few days [1], [27]. According to the review by Oviedo-Boyso et al. [1], in the present study, *S. aureus*-induced mRNA expression of IL-1β and TNF-α was increased at least after 6 h of stimulation and decreased again until 24 h post challenge. The observation that SLAT(+) and SLAT(−) strains induced a different expression of these pro-inflammatory cytokines in bMEC may indicate that these strains induce different severities of mastitis. However, the severity of mastitis that these strains induced is not known.

The stronger induction of the mRNA expression pattern of the chemokines IL-8 and RANTES in bMEC may suggest earlier and stronger leukocyte recruitment during the innate immune response after an intramammary infection with SLAT(+) compared to infections with SLAT(−) strains. The immediate recruitment of somatic cells from the blood into the udder is essential for effective elimination of intramammary pathogens [28]. Thus, a deviating time point and magnitude of leukocyte recruitment, which is reflected in the chemokine expression, might influence the clinical course of mastitis. Since no clinical data including somatic cell count were available for the tested *S. aureus* strains in the present study, conclusions cannot be drawn on the clinical manifestation of the infection.

The acute phase protein SAA is known to be expressed in bMEC and is upregulated during mastitis [29]. Wellnitz et al. [21] reported similarly increased mRNA levels of SAA in cells challenged for 6 h with *S. aureus* or *E. coli*. In the present study, SLAT(+) induced a stronger SAA mRNA expression compared to SLAT(−) *S. aureus* strains. However, mRNA levels induced by *E. coli* were equal or higher during the whole study than in response to *S. aureus*, depending on the SLAT type. As SAA is also a chemoattractant [30], [31], pathogens that upregulate SAA transcription could be associated with an increase of SCC in the mammary gland. This effect could, as described for IL-8 above, have an effect on the progress of the mastitis.

Epithelial cells from the mammary gland are the major source of the iron-binding protein Lf that increases in milk during bovine clinical mastitis [28]. Griesbeck-Zilch et al. [12] showed a more pronounced Lf mRNA expression in bMEC by *S. aureus* than by *E. coli* challenge. In the present study, *E. coli* induced comparable Lf mRNA levels to SLAT(+) but higher levels compared to SLAT(-). However, neither the SLAT phenotype of *S. aureus* strains, nor details on the *E. coli* strain used in the study of Griesbeck-Zilch et al. [12] are known, which could account for a deviating Lf expression pattern in bMEC. Comparing the Lf mRNA expression between *S. aureus* strains in the present study clearly shows that SLAT(+) strains induced higher levels than SLAT(-), which again confirms a deviating immune response of the mammary gland to SLAT(+) and SLAT(−) strains. Only SLAT(−) strain *1904* caused Lf mRNA levels that were not significantly lower than in response to two of the SLAT(+) strains. Thus, the induction of Lf mRNA expression in bMEC might follow different strain-dependent mechanisms.

Another cytokine that was stronger induced on the mRNA level by SLAT(+) compared to SLAT(−) *S. aureus* strains in bMEC is GM-CSF. Since GM-CSF is responsible for an appropriate supply of leukocytes, the effector cells of the innate immune system, it is likely that high levels of GM-CSF expression represent a strong inflammatory response in the mammary gland. As a consequence, it is possible that SLAT(+) cause more severe forms of mastitis.

The transient upregulation of GM-CSF in response to all SLAT(+), as well as to the majority of SLAT(−) strains indicates that the role of bMEC in stimulating phagocyte differentiation is rather short term. This is consistent with an in vivo study, where mRNA expression of somatic cells upon *S. aureus* infection was evaluated [32].

The observation that TLR2 mRNA, a receptor for cell wall components of gram-positive bacteria, was induced in response of the majority of the *S. aureus* strains, confirms the involvement of this pattern recognition receptor (PRR) [12]. SLAT(+) *S. aureus* strains showed a stronger induction of TLR2 mRNA than SLAT(−) strains. Interestingly, one strain, SLAT(−) *1586*, apparently did not induce TLR2 mRNA expression in bMEC. One possible reason could be that this strain has an altered PAMP, i.e. LTA or peptidoglycan that is normally recognized by this PRR. To confirm this, further investigations are necessary. Even though most studies describe TLR2 as a primary receptor for gram-positive bacteria, it is also known that this PPR is responsive to gram-negative bacteria [33]. This point also explains the induced TLR2 expression by *E. coli*.

Prostaglandins are further inflammatory mediators in the bovine mammary gland with chemotactic activity [34]. COX-2 is one of the enzymes involved in prostaglandin synthesis that is transiently upregulated during inflammation [35], [36]. These findings are reflected by this study. COX-2 was only transiently upregulated in bMEC in response to all *S. aureus* strains, except to one SLAT(−) strain. Compared to the other measured immune factors, the SLAT(+) group is not as strong of an inducer of COX-2 and TLR2 compared to SLAT(−), since differences were not consistently significant between the SLAT groups.

## Conclusion

The results of this study clearly indicate a different immunological response of bMEC to SLAT(+) and SLAT(−) strains. Although individual differences within SLAT groups and the cow seem to influence the immune response, SLAT(+) *S. aureus* strains induce a more pronounced transcription of several important immune factors compared to SLAT(−) strains in mammary epithelial cells. Furthermore, results obtained from adhesion assays indicate that SLAT(+) *S. aureus* strains show an increased affinity to adhere to bMEC than SLAT(−) strains. These findings together with molecular data on the *S. aureus* strains might support the hypothesis that SLAT(+) exceed SLAT(−) strains in virulence. The influence of these effects on the varying course and severity of mastitis can be suggested and should be further investigated in vivo.
